# Relation between air pollution and allergic rhinitis in Taiwanese schoolchildren

**DOI:** 10.1186/1465-9921-7-23

**Published:** 2006-02-09

**Authors:** Bing-Fang Hwang, Jouni JK Jaakkola, Yung-Ling Lee, Ying-Chu Lin, Yue-liang Leon Guo

**Affiliations:** 1 School and Graduate Institute of Occupational Safety and Health, College of Public Health, China Medical University, 91, Hsueh-Shih Road, Taichung, 40402, Taiwan; 2 Department of Environmental and Occupational Health, College of Medicine, National Cheng Kung University, 138 Sheng-Li Road, Tainan 704, Tainan, Taiwan; 3 Department of Health Care Administration, Diwan College of Management, 87-1, Nansh Li, Madou Jen, Tainan 721, Taiwan; 4 Institute of Occupational and Environmental Medicine, The University of Birmingham, Edgbaston, Birmingham B15 2TT, UK; 5 Department of Internal Medicine, National Cheng Kung University Hospital, 138 Sheng-Li Road, Tainan 704, Taiwan; 6 College of Dental Medicine, Kaohsiung Medical University, 100 Shi-Chuan 1st Road, San Ming District, Kaohsiung City, Taiwan; 7 Department of Environmental and Occupational Medicine, National Taiwan University, Taipei 100, Taiwan

## Abstract

**Background:**

Recent findings suggest that exposure to outdoor air pollutants may increase the risk of allergic rhinitis. The results of these studies are inconsistent, but warrant further attention. The objective of the study was to assess the effect of relation between exposure to urban air pollution and the prevalence allergic rhinitis among school children.

**Methods:**

We conducted a nationwide cross-sectional study of 32,143 Taiwanese school children. We obtained routine air-pollution monitoring data for sulphur dioxide (SO_2_), nitrogen oxides (NOx), ozone (O_3_), carbon monoxide (CO), and particles with an aerodynamic diameter of 10 μm or less (PM_10_). A parent-administered questionnaire provided information on individual characteristics and indoor environments (response rate 92%). Municipal-level exposure was calculated using the mean of the 2000 monthly averages. The effect estimates were presented as odds ratios (ORs) per 10 ppb change for SO_2_, NOx, and O_3_, 100 ppb change for CO, and 10 μg/m^3 ^change for PM_10_.

**Results:**

In two-stage hierarchical model adjusting for confounding, the prevalence of allergic rhinitis was significantly associated with SO_2 _(adjusted odds ratio (OR) = 1.43, 95% confidence interval (CI): 1.25, 1.64), CO (aOR = 1.05, 95% CI: 1.04, 1.07), and NOx (aOR = 1.11, 95% CI: 1.08, 1.15). Contrary to our hypothesis, the prevalence of allergic rhinitis was weakly or not related to O_3 _(aOR = 1.05, 95% CI: 0.98, 1.12) and PM_10 _(aOR = 1.00, 95% CI: 0.99, 1.02).

**Conclusion:**

Persistent exposure to NOx, CO, and SO_2 _may increase the prevalence of allergic rhinitis in children.

## Background

The prevalence of allergic rhinitis is increasing among children in many countries [1]. There is accumulating evidence that both genetic and environmental factors play important roles in the aetiology of allergic rhinitis. It is likely that there is a multilevel interaction between genetic and environmental factors [2]. Changes in genetic pool are an unlikely to explain changes in the occurrence of allergic rhinitis on short time interval. Therefore, attempts to identify environmental factors are useful for prevention [3]. Identification of indicators for genetic susceptibility to environmental exposures could also be useful from preventive point of view. Recent findings suggest that exposure to outdoor air pollutants may increase the risk of allergic rhinitis in children [4-9]. The results of these studies are inconsistent, but warrant further attention.

In 1995–1996, Lee et al. studied the association between air pollution and allergic rhinitis in Taiwan. This study of 331,686 children showed a relation between the risk of allergic rhinitis and a score of traffic-related air pollutants derived from municipal concentrations of carbon monoxide (CO) and nitrogen oxides (NOx) [10]. Relations between the prevalence of allergic rhinitis and the concentrations of individual pollutants were not studied. This study was not able to adjust for parental atopy or indoor exposures, which are potential sources of confounding and effect modification.

In 2001, we conducted a new nationwide cross-sectional study, where we collected information also on those important potential determinants of allergic disease in children. Our primary objective was to assess the relation between exposure to urban air pollution and the prevalence of allergic rhinitis in schoolchildren, focussing on predominantly traffic-related pollutants such as nitrogen oxides (NOx), ozone (O_3_), carbon monoxide (CO), pollutants from other fossil fuel combustion sources, such as sulphur dioxide (SO_2_), and particles with an aerodynamic diameter of 10 μm or less (PM_10_). In addition, we hypothesised that the joint effect of parental atopy and exposure to outdoor air pollution on prevalence of the allergic rhinitis is more than the expected on the basis of their independent effects. We assumed that parents with asthma, allergic rhinitis or allergic atopic eczema may give their children genes that increase the susceptibility to the effects of environmental factors on allergic rhinitis.

## Methods

### Data collection and study population

In 2001, we conducted a nationwide cross-sectional study in Taiwan using a modified Chinese version of The International Study of Asthma and Allergies in Childhood (ISAAC-C) questionnaire [11]. The questionnaire inquired details of children's health, environmental exposures, and other relevant information. The study population was recruited from elementary and middle schools in 22 municipalities within one kilometre from a Taiwan Environmental Protection Agency (EPA) air-monitoring station. First, we randomly selected one monitoring station in each county. We then randomly selected one school next to each monitoring station. Finally, we conducted a stratified sampling of the students by selecting 5–7 classes per grade from each school. The questionnaires were taken home by students and answered by parents. A total of 35,036 children aged 6–15 years were approached. The response rate was 91.7%. We excluded 2,893 children because of incomplete questionnaire and personal history of atopic ecezma. Therefore, the final study population included 32,143 school children. The study protocol was approved by the Respiratory Health Screening Steering Committee of the Taiwan Department of Health and the Institutional Review Board at the National Cheng Kung University Hospital, and it complied with the principles outlined in the Helsinki Declaration [12].

### Health outcome

The outcome of interest was allergic rhinitis, which was defined on the basis of answers to the question: "Has a physician ever diagnosed your child as having allergic rhinitis?" (yes; no). The questionnaire also included a question on the symptoms of allergic rhinitis per se. After primary analyses, we decided to focus on physician-diagnosed allergic rhinitis.

Physician-diagnosed allergic rhinitis reflects well the occurrence of allergic rhinitis, because Taiwanese children are almost all covered by health insurance (>99%) and there is a good access to health care. Thus children with allergic rhinitis are commonly diagnosed. A history of atopic eczema was defined as the presence of itching skin eruption at cubital, posterior popliteal, neck, periauricle, and eyebrow areas for 6 months or longer and a diagnosis of atopic eczema by physician.

### Exposure assessment

Monitoring data for sulphur dioxide (SO_2_), nitrogen oxides (NOx), ozone (O_3_), carbon monoxide (CO), particles with an aerodynamic diameter of 10 μm or less (PM_10_), as well as for temperature and relative humidity, are available from Taiwan Environmental Protection Agency in 1994 and later years. Concentrations of each pollutant are measured continuously and reported hourly – CO by non-dispersive infrared absorption, NOx by chemiluminescence, O_3 _by ultraviolet absorption, SO_2 _by ultraviolet fluorescence, and PM_10 _by beta-gauge.

Exposure parameters in the present study were annual averages of air pollutants, calculated from the monthly averages of the year 2000. Exposure assessment was performed for children attending schools located within one km of 22 of these monitoring stations.

### Covariates

Information on potential confounders was obtained from the questionnaire. The covariates in the present analyses included age, gender, parental atopy, parental education, maternal smoking during pregnancy, and environmental tobacco smoke (ETS), cockroaches noted monthly, water damage and visible mould in the home (Table 1). Parental atopy was a measure of genetic predisposition and was defined as the father or the mother of the index child ever having been diagnosed as having asthma, allergic rhinitis, or atopic eczema.

**Table 1:** Number of children with allergic rhinitis, and prevalence of allergic rhinitis with 95% confidence interval (95% CI) by selected covariates in Taiwan 2001.

Determinant	No. of children	No. of physician- diagnosis allergic rhinitis	Prevalence (P%)	OR (95% CI)
Total	32,143	8,202	25.5	
Age (years)				
< = 7	4,589	1,255	27.3	1.46 (1.30–1.65)
8	3,483	987	28.3	1.54 (1.36–1.74)
9	3,495	972	27.8	1.50 (1.32–1.70)
10	3,695	1,006	27.2	1.45 (1.29–1.65)
11	3,478	931	26.8	1.42 (1.25–1.61)
12	3,749	886	23.6	1.20 (1.06–1.36)
13	3,697	867	23.5	1.19 (1.05–1.35)
14	3,611	818	22.7	1.14 (1.00–1.29)
15	2,346	480	20.5	1.00
Gender				
Male	16,277	4,970	30.5	1.72 (1.63–1.81)
Female	15,866	3,232	20.4	1.00
Parental education (years)				
<6	1,789	267	14.9	1.00
6–8	5,603	1,056	18.8	1.32 (1.14–1.53)
9–11	14,492	3,643	25.1	1.91 (1.67–2.19)
> = 12	10,259	3,236	31.5	2.63 (2.29–3.01)
Parental atopy				
Yes	9,143	4,356	47.6	4.59 (4.35–4.85)
No	22,499	3,721	16.5	1.00
Environmental tobacco smoke§				
Yes	18,861	4,512	23.9	0.82 (0.78–0.86)
No	13,053	3,632	27.8	1.00
Maternal smoking during pregnancy§				
Yes	689	140	20.3	0.74 (0.61–0.89)
No	31,292	8,027	25.7	1.00
Cockroaches noted monthly§				
Yes	25,113	6,557	26.1	1.09 (0.99–1.20)
No	6,548	1,547	23.6	1.00
Water damage§				
Yes	2,614	629	24.1	0.92 (0.84–1.01)
No	29,383	7,547	25.7	1.00
Visible mould§				
Yes	7,469	2,258	30.2	1.37 (1.29–1.45)
No	23,923	5,764	24.1	1.00

§Numbers of subjects do not add up to total N because of missing data.

### Statistical methods

We applied two-stage hierarchical models, which allowed an appropriate adjustment for confounding and effect modification on individual-level and assessment of the effects of air pollution on municipal-level [13,14]. We used odds ratio as a measure of the relation between exposure to air pollution and the prevalence of allergic rhinitis. We estimated adjusted odds ratios in a two-stage hierarchical model using logistic and linear regression analyses. The detail was described elsewhere [15]. The results from the models are presented as odds ratios (ORs), along with their 95% confidence intervals (CIs). First, we fitted single-pollutant models estimating the increase in adjusted log odds per increase in air pollutant level (Table 4). We then considered two-pollutant models by fitting one traffic-related and one stationary fossil fuel combustion-related pollutant. Finally, we also fitted two-pollution models with O_3 _and another pollutant. The two-pollutant models provide estimates of the independent effects of CO, NOx, SO_2_, PM_10_, and O_3 _on allergic rhinitis controlling for the other pollutant in the model. The effect of each pollutant on the prevalence of allergic rhinitis was presented as odds ratios (ORs) per 10 ppb change for SO_2_, NOx, and O_3_, 100 ppb change for CO, and 10 μg/m^3 ^change for PM_10_, along with their 95% confidence intervals (CIs). The goodness of fit was assessed with likelihood ratio tests (LR) to determine whether a variable contributed significantly to the model.

## Results

### Study population and occurrence of allergic rhinitis

Table 1 displays the characteristics of the study population and the prevalence of allergic rhinitis according to the covariates. The overall prevalence of allergic rhinitis was estimated as 25.5% (95% CI: 25.0%, 26.0%). The prevalence of allergic rhinitis was positively associated with age, higher parental education level, male gender, and parental atopy. The prevalence was also related to the presence of cockroaches, although not statistically significantly. There was an association with visible mould in home but not with water damage. In contrast, a negative association was found for environmental tobacco smoke (ETS) and maternal smoking during pregnancy.

### Air pollution

Table 2 'see additional file 1' summarizes the distributions of the annual mean air pollutant concentrations, temperature and relative humidity in the 22 monitoring stations in the year 2000. The correlations between different pollutants are shown in Table 3 'see additional file 2'. The correlation structure is generally consistent with the common sources of the traffic-related pollutants (CO, and NOx) and stationary fossil fuel combustion-related pollutants (SO_2_, and PM_10_). The correlation between NOx and CO concentrations was high (0.88), which reflects motor vehicles as the common source. The high correlation also implied that only one of the two pollutants could be used as an indicator of traffic-related pollution in the models estimating effects on the prevalence of allergic rhinitis. The correlation of PM_10 _and SO_2 _concentrations was also relatively high (0.58) indicating stationary fuel combustion as the common source, although SO_2 _concentrations were also correlated with both traffic-related pollutants. The concentrations of O_3 _were negatively correlated with the mainly traffic-related pollutants, but positively with PM_10 _and SO_2_, and it was only weakly correlated with those of traffic-related and stationary fossil fuel combustion-related air pollutants.

### Air pollution and allergic rhinitis

The prevalence of allergic rhinitis was consistently related to the levels of traffic-related pollutants. In the single-pollutant model, the adjusted odds ratio for 10 bbp change in NOx was 1.11 (95% CI 1.08–1.15), and the estimate changed little when a second pollutant was added (Table 4: Models 1–3). The adjusted odds ratio for 100 ppb change in CO was 1.05 (95% CI 1.04–1.07) and again addition of SO_2 _(1.04), PM_10 _(1.05), or O_3 _(1.07) had little influence (Table 4 'see additional file 3' : Models 4, 5 and 6). The adjusted odds ratio for 10 ppb change in SO_2 _alone was 1.43 (95% CI 1.25–1.64), but inclusion of either of the traffic-related pollutants reduced the effect estimate substantially (Table 4 'see additional file 3' : Models 1 and 4), whereas addition of O_3 _had little influence (Table 4 'see additional file 3' : Model 7). The prevalence of allergic rhinitis was not related to PM_10 _concentrations in any combination of air pollutants (Table 4 'see additional file 3' : Models 2, 5 and 8). In the single-pollutant model, there was no significant association between O_3 _and the prevalence of allergic rhinitis, but an addition of either NOx or CO resulted in elevated, statistically significant effect estimates (Table 4 'see additional file 3' : Models 3 and 6).

In summary, positive statistically significant associations were found for SO_2_, and traffic-related pollutants (CO and NOx). In contrast, negative or weak associations were found for O_3 _and PM_10_.

In order to elaborate the residual confounding and potential effect modification, we systematically conducted stratified analyses in different categories of gender, parental atopy, parental education, and presence of exposure to ETS and visible moulds in the home. The stratified analyses did not indicate any major residual confounding or effect modification (Table 5 'see additional file 4').

## Discussion

In our nationwide cross-sectional study of Taiwanese school children, the prevalence of allergic rhinitis was statistically significantly associated with annual levels of the two traffic-related pollutants, NOx and CO, as well as SO_2_. The prevalence of allergic rhinitis was inconsistently related to levels of O_3 _and consistently not related to levels of PM_10_.

Furthermore, the results did not provide evidence that the joint effect of hereditary atopy representing genetic predisposition and outdoor air pollutants exposure is stronger than expected on the basis of their independent effects.

### Validity of results

The exposure assessment was based on routine air-pollution monitoring data. The monitoring data represented reasonably well exposures both in the school and in the home for two reasons. The schools were chosen to be near the monitoring stations. Almost all the children attended schools within one kilometre of their homes, because the density of elementary and middle schools in Taiwan is very high. Finally, the two-stage hierarchical modelling took into account the fact that municipal-level exposure information was used.

The cross-sectional study design is susceptible to selection bias. Parents of children with respiratory problems linked to air pollution could move to residential areas with lower levels of air pollution, which would lead to underestimation of the exposure-outcome relations. Any random migration was likely to result in underestimation of the air pollution effects rather than introducing a positive bias in the associations. Information on residential history in a cross-sectional study or a longitudinal study design is needed to minimise this potential bias.

We were able to adjust for a number of potential individual-level confounders such as parental atopy and education and central indoor environmental exposures. We also elaborated the possibility of residual confounding by studying the relations of interest in different levels of covariates. Parental education had a positive association with concentrations of traffic-related pollutants. Also the prevalence of allergic rhinitis was positively associated with the level of parental education (Table 1). Thus parental education was a potential confounder of the relations between air pollution levels and the risk of allergic rhinitis. To elaborate this, we assessed the relation between air pollution levels and the prevalence of allergic rhinitis on different levels of parental education, and showed that the stratum-specific relations were relatively consistent (Table 5 'see additional file 4'), which reassured that parental education did not act as a confounder.

Urban air pollution constitutes a complex mixture of several compounds and the assessment of the independent effects of different pollutants is a major challenge, which includes both the issues of confounding and effect modification (joint effect of several compounds). The correlations between different compounds are consistent with our knowledge of the sources of air pollution. NOx and CO concentrations were highly correlated representing motor vehicle traffic, whereas SO_2 _and PM_10 _concentrations were more related to other combustion sources. In the modelling, it was feasible to control for stationary fossil fuel pollutants as a potential confounder when assessing the effects of traffic-related pollutants and vice versa.

However, due to collinearity problems, it was not possible to separate the effects of traffic-related pollutants from each other (NOx and CO).

### Synthesis with previous knowledge

The results of the present study and one previous study from Germany [5], are consistent with the hypothesis that long-term exposure to outdoor air pollutants increases the risk of allergic rhinitis in children. Both studies suggest an increased risk related to traffic-related air pollutants (NOx). In a British study the occurrence of general practise consultations due to allergic rhinitis was related to short-term exposure to SO_2 _and O_3_. The strongest associations were found for daily levels during 3 to 4 days prior to consultation [6].

Few air pollution studies have addressed allergic rhinitis as an outcome among children. A German study provided little evidence that exposure to high concentration of SO_2_, and moderate levels of NOx, and PM_10 _was related to the occurrence of upper respiratory symptoms, including runny nose, cough and hoarseness [4]. Another German study indicated that the prevalence of symptoms of allergic rhinitis is related to traffic-related outdoor air pollutants (NO_2_) [5]. No association between prevalence of allergic rhinitis and mean SO_2_, NO_2 _and O_3 _was identified in French ISAAC study [7]. A cross-sectional study in Germany found no association between traffic-related air pollutants and prevalence of atopic symptoms [8]. Another survey conducted in French primary school children reported that the prevalence of atopy was not related to the levels of photochemical air pollutants [9].

Nitrogen dioxide has been shown to be an acute respiratory irritant in controlled exposure studies [16]. There are no plausible mechanisms through which CO exposure would influence the airways and increase the risk of allergic rhinitis. Both NOx and CO represent the complex mixture of traffic exhaust, and NO_2 _is known to be the best indicator of motor vehicle traffic emissions. In the present study, it was not possible to elaborate to what extent NOx would have direct effects on children airways. CO is unlikely to have any direct effects on the respiratory tract. Our finding of a lack of association between the risk of allergic rhinitis and PM_10 _levels is consistent with the results from the Harvard 24 Cities Study in North America [17]. Although the risk of allergic rhinitis was not related to the levels of PM_10_, it is likely that there is an association with fine particulate matter (PM 2.5) and ultrafine particles typically present in motor vehicle exhausts and in particular in diesel exhausts, which can enhance allergic inflammation and induce the development of allergic immune responses. Further studies should assess these relations.

A positive association between the risk of allergic rhinitis and SO_2 _levels was identified, compatible with a toxicological study [18]. SO_2 _may increase the permeability of the mucous membrane in airways, which may favour the penetration of allergens and the development of allergic reactions. High traffic density is inversely related to concentrations of ozone (O_3_) [19], which is formed at some distance from emission sources and scavenged in city centres by nitrogen monoxide (NO) from vehicle exhaust. The concentrations of O_3 _were negatively correlated with the mainly traffic-related pollutants (Table 3 'see additional file 2'). The prevalence of allergic rhinitis was associated with the levels of O_3 _only when adjusting for a traffic-related pollutant. This is consistent with the hypothesis that the direct emissions from motor vehicles, which scavenge O_3 _and therefore are negatively associated with O_3_, are more important determinants of prevalence of allergic rhinitis than the secondary pollutants, such as O_3_, that are formed downwind. O_3 _is a known respiratory irritant [20] and could also influence the permeability of the airways mucous membranes contributing to allergic rhinitis.

According to epidemiologic and toxicologic evidence, the World Health Organization (WHO) concluded that traffic related air pollution may increase the risk of allergic development and exacerbate symptoms in particular in susceptible subgroups [21]. Traffic related air pollutants may also increase the risk of non-allergic respiratory symptoms and disease due to their irritative properties [22]. The recent epidemiologic studies suggested that the evidence of the effect of persistent exposure to air pollution on allergic rhinitis still is weak and inconclusive [4-9].

## Conclusion

The present study showed statistically significant relations between exposure to outdoor air pollutants and the prevalence of allergic rhinitis in schoolchildren. The observed relations of the risk of allergic rhinitis to NOx and CO levels suggest that emissions from motor vehicles play an important role. In addition, the relation to SO_2 _levels indicates that also other combustion of fossil fuels contribute to adverse health effects.

## List of abbreviations used

NOx, nitrogen oxides

PM_10_, particles with aerodynamic diameter 10 μm or less

SO_2_, sulphur dioxide

O_3_, ozone

CO, carbon monoxide

ppb, part per billion

## Competing interests

The author(s) declare that have no competing interests.

## Authors' contributions

Bing-Fang Hwang is responsible for obtained funding, study concept and design, integrity of the data, the accuracy of the data analysis, and drafting of the manuscript; Jouni JK Jaakkola for planning of the statistical analyses and critical revision of the manuscript for important intellectual content; Yung-Ling Lee for data management, data collection, and manuscript comments; Ying-Chu Lin for data collection and manuscript comments; Yueliang Leo Guo for obtained funding, study concept and design, and study supervision. All authors read and approved the final manuscript.

## Supplementary Material

Additional File 1 Table 2. Annual air pollution and meteorological data from 22 monitoring stations in Taiwan, 2000. Click here for file

Additional File 2 Table 3. Correlations between air pollutants across 22 municipalities. Click here for file

Additional File 3 Table 4. Adjusted odds ratios (ORs), along with 95% confidence interval (CIs) of physician-diagnosis allergic rhinitis in single and two pollutant models. Click here for file

Additional File 4 Table 5. Adjusted odds ratios (ORs), along with 95% confidence interval (CIs) of physician-diagnosis allergic rhinitis stratified by different levels of covariates in the relation between allergic rhinitis and air pollutants. Click here for file
