# Supplementary material for: Relation between air pollution and allergic rhinitis in Taiwanese schoolchildren
Source: Respir Res. 2006 Feb 9;7(1):23. doi: 10.1186/1465-9921-7-23 (PMC1420289; doi:10.1186/1465-9921-7-23)
Supplement: Additional File 1 — Table 2. Annual air pollution and meteorological data from 22 monitoring stations in Taiwan, 2000. [file 1465-9921-7-23-S1.pdf]

**Table 2:** Annual air pollution and meteorological data from 22 monitoring stations in Taiwan, 2000

| | Mean±SD | Minimum | 25th percentile | Median | 75th percentile | Interquartile range[a] | Maximum |
|---|---|---|---|---|---|---|---|
| CO (ppb) | 664±153 | 416 | 540 | 647 | 752 | 212 | 964 |
| NOx (ppb) | 27.64±8.38 | 10.88 | 21.47 | 29.58 | 33.07 | 11.60 | 43.98 |
| $O_3$ (ppb) | 23.14±3.25 | 18.65 | 20.34 | 22.69 | 25.20 | 4.86 | 31.17 |
| $PM_{10}$ (µg/m$^3$) | 55.58±16.57 | 29.36 | 42.96 | 53.81 | 70.37 | 27.41 | 99.58 |
| $SO_2$ (ppb) | 3.53±2.00 | 0.35 | 1.96 | 3.34 | 4.84 | 2.88 | 10.12 |
| Temperature (˚C) | 23.65±0.75 | 22.36 | 23.20 | 23.50 | 24.17 | 0.97 | 25.27 |
| Relative humidity | 74.0±3.0% | 69.0% | 72.2% | 74.1% | 76.5% | 4.3% | 80.0% |

Abbreviations: NOx, nitrogen oxides; $PM_{10}$, particles with aerodynamic diameter 10 µm or less; $SO_2$, sulphur dioxide; $O_3$, ozone; CO, carbon monoxide; ppb, part per billion.
[a] Range form 25th to 75th percentile of site-specific concentrations.
