# Supplementary material for: Relation between air pollution and allergic rhinitis in Taiwanese schoolchildren
Source: Respir Res. 2006 Feb 9;7(1):23. doi: 10.1186/1465-9921-7-23 (PMC1420289; doi:10.1186/1465-9921-7-23)
Supplement: Additional File 2 — Table 3. Correlations between air pollutants across 22 municipalities. [file 1465-9921-7-23-S2.pdf]

Table 3: Correlations between air pollutants across 22 municipalities.

	CO	NOx	O_3	PM_{10}	SO_2
CO	1.00	0.88*	-0.37	0.27	0.40
NOx		1.00	-0.39	0.34	0.50*
O_3			1.00	0.28	0.19
PM_{10}				1.00	0.58*
SO_2					1.00

Abbreviations: NOx, nitrogen oxides; PM_{10}, particles with aerodynamic diameter 10 µm or less; SO_2, sulphur dioxide; O_3, ozone; CO, carbon monoxide.
* Correlation is significant at the 0.05 level
