# Supplementary material for: Relation between air pollution and allergic rhinitis in Taiwanese schoolchildren
Source: Respir Res. 2006 Feb 9;7(1):23. doi: 10.1186/1465-9921-7-23 (PMC1420289; doi:10.1186/1465-9921-7-23)
Supplement: Additional File 3 — Table 4. Adjusted odds ratios (ORs), along with 95% confidence interval (CIs) of physician-diagnosis allergic rhinitis in single and two pollutant models. [file 1465-9921-7-23-S3.pdf]

Table 4: Adjusted odds ratios (ORs), along with 95% confidence interval (CIs) of physician-diagnosis allergic rhinitis in single and two pollutant models.

	Single-pollutant model	Two-pollutant model 1 (NOx+SO_2)	Two-pollutant model 2 (NOx+PM_{10})	Two-pollutant model 3 (NOx+O_3)	Two-pollutant model 4 (CO+SO_2)	Two-pollutant model 5 (CO+PM_{10})	Two-pollutant model 6 (CO+O_3)	Two-pollutant model 7 (SO_2+O_3)	Two-pollutant model 8 (PM_{10}+O_3)
NOx	1.11	1.10	1.13	1.14					
(10ppb)	(1.08-1.15)	(1.06-1.14)	(1.09-1.17)	(1.10-1.18)					
CO	1.05				1.04	1.05	1.07		
(100 ppb)	(1.04-1.07)				(1.02-1.06)	(1.03-1.07)	(1.05-1.09)		
SO_2	1.43	1.14			1.26			1.43	
(10 ppb)	(1.25-1.64)	(0.98-1.31)			(1.09-1.45)			(1.24-1.65)	
PM_{10}	1.00		0.99			1.00			1.00
(10 $\mu g/m^3$)	(0.99-1.02)		(0.97-1.00)			(0.99-1.01)			(0.99-1.02)
O_3	1.05			1.20			1.18	1.04	1.08
(10 ppb)	(0.98-1.12)			(1.10-1.32)			(1.07-1.29)	(0.95-1.14)	(0.99-1.18)

* Two-stage hierarchical analysis adjusting for age, gender, parental education, parental atopy, environmental tobacco smoke (ETS), and visible mould.

Abbreviations: NOx, nitrogen oxides; PM_{10}, particles with aerodynamic diameter 10 µm or less; SO_2, sulphur dioxide; O_3, ozone; CO, carbon monoxide; ppb, part per million.
