# Supplementary material for: Relation between air pollution and allergic rhinitis in Taiwanese schoolchildren
Source: Respir Res. 2006 Feb 9;7(1):23. doi: 10.1186/1465-9921-7-23 (PMC1420289; doi:10.1186/1465-9921-7-23)
Supplement: Additional File 4 — Table 5. Adjusted odds ratios (ORs), along with 95% confidence interval (CIs) of physician-diagnosis allergic rhinitis stratified by different levels of covariates in the relation between allergic rhinitis and air pollutants. [file 1465-9921-7-23-S4.pdf]

Table 5: Adjusted odds ratios (ORs), along with 95% confidence interval (CIs) of physician-diagnosis allergic rhinitis stratified by different levels of covariates in the relation between allergic rhinitis and air pollutants.

	Gender	aOR (95% CI) *	Parental atopy	aOR (95% CI) *ƒ*	Parental education	aOR (95% CI)†	ETS	aOR (95% CI) §	Visible mould	aOR (95% CI) ¶
NOx (10ppb)	Male	1.12 (1.07-1.16)	Yes	1.05 (1.02-1.08)	<6 years	1.01 (0.85-1.21)	Yes	1.06 (1.03-1.08)	Yes	1.07 (1.03-1.11)
	Female	1.12 (1.06-1.17)	No	1.06 (1.03-1.08)	6-8 years	1.16 (1.06-1.28)	No	1.05 (1.02-1.08)	No	1.05 (1.03-1.07)
					9-11 years	1.10 (1.04-1.15)				
					>=12 years	1.13 (1.07-1.19)				
CO (100 ppb)	Male	1.06 (1.03-1.08)	Yes	1.05 (1.02-1.08)	<6 years	1.00 (0.91-1.09)	Yes	1.06 (1.03-1.08)	Yes	1.07 (1.03-1.11)
	Female	1.05 (1.02-1.08)	No	1.06 (1.03-1.08)	6-8 years	1.07 (1.02-1.12)	No	1.05 (1.02-1.08)	No	1.05 (1.03-1.07)
					9-11 years	1.05 (1.02-1.08)				
					>=12 years	1.06 (1.03-1.09)				
SO_2 (10 ppb)	Male	1.61 (1.34-1.93)	Yes	1.34 (1.08-1.67)	<6 years	1.48 (0.76-2.89)	Yes	1.33 (1.11-1.59)	Yes	1.61 (1.22-2.13)
	Female	1.26 (1.02-1.56)	No	1.52 (1.27-1.81)	6-8 years	1.36 (0.96-1.94)	No	1.64 (1.32-2.04)	No	1.40 (1.19-1.64)
					9-11 years	1.35 (1.10-1.66)				
					>=12 years	1.62 (1.28-2.06)				
PM_{10} (10 $\mu g/m^3$)	Male	1.02 (0.99-1.04)	Yes	1.00 (0.98-1.03)	<6 years	1.05 (0.96-1.14)	Yes	1.01 (0.99-1.03)	Yes	1.02 (0.99-1.06)
	Female	0.99 (0.97-1.02)	No	1.01 (0.99-1.03)	6-8 years	1.03 (0.98-1.07)	No	1.00 (0.98-1.03)	No	1.00 (0.98-1.02)
					9-11 years	1.00 (0.98-1.03)				
					>=12 years	0.99 (0.97-1.02)				
O_3 (10 ppb)	Male	1.07 (0.95-1.20)	Yes	1.03 (0.90-1.18)	<6 years	1.06 (0.74-1.50)	Yes	1.07 (0.95-1.19)	Yes	1.20 (1.01-1.42)
	Female	1.10 (0.96-1.25)	No	1.12 (1.00-1.25)	6-8 years	1.07 (0.89-1.30)	No	1.11 (0.97-1.28)	No	1.05 (0.95-1.16)
					9-11 years	1.12 (0.98-1.27)				
					>=12 years	1.05 (0.89-1.24)				

* Two-stage hierarchical analysis adjusting for age, parental education, parental atopy, environmental tobacco smoke (ETS), and visible mould.

†Two-stage hierarchical analysis adjusting for age, gender, parental atopy, environmental tobacco smoke (ETS), and visible mould.

ƒ Two-stage hierarchical analysis adjusting for age, gender, parental education, environmental tobacco smoke (ETS), and visible mould.

§Two-stage hierarchical analysis adjusting for age, gender, parental education, parental atopy, and visible mould.

¶ Two-stage hierarchical analysis adjusting for age, gender, parental education, parental atopy, and environmental tobacco smoke (ETS).

Abbreviations: NOx, nitrogen oxides; PM_{10}, particles with aerodynamic diameter 10 µm or less; SO_2, sulphur dioxide; O_3, ozone; CO, carbon monoxide; ppb, part per billion.
